# HOW ARE FAMILIES INVOLVED IN THE CARE OF HOSPITALIZED OLDER ADULTS? A QUALITATIVE EVIDENCE SYNTHESIS

**DOI:** 10.1093/geroni/igae098.2228

**Published:** 2024-12-31

**Authors:** Judith Vick, Connor Fogleman, Joseph Neiman, Sarah Cantrell, Mollie Selmanoff, Tolu Oyesanya, Karen Goldstein, Courtney Van Houtven

**Affiliations:** Yale University School of Medicine, New Haven, Connecticut, United States; Duke University, Durham, North Carolina, United States; Durham VA Health Care System, Durham, North Carolina, United States; Duke University, Durham, North Carolina, United States; Johns Hopkins Hospital, Baltimore, Maryland, United States; Duke University School of Medicine, Durham, North Carolina, United States; Duke University School of Medicine, Durham, North Carolina, United States; Duke University School of Medicine, Durham, North Carolina, United States

## Abstract

In the outpatient setting, roughly 36 million family members in the US assist with caregiving tasks for older adults including managing medications, coordinating care, communicating with clinicians, and assisting with activities of daily living. The roles and impact of family involvement during hospitalizations of older adults are less well described. To better understand family involvement during hospitalizations of older adults, we designed a systematic review of qualitative research known as a qualitative evidence synthesis. We assembled a multidisciplinary research team including members with expertise in nursing, internal medicine, library science, evidence synthesis, social work, and health services research. We developed a search strategy and executed it in four databases: MEDLINE (via Ovid), Embase (via Elsevier), PsycINFO (via Ovid), and CINAHL Complete (via EBSCO). Our search identified 6,157 articles, and our title/abstract screening yielded 157 articles for full text review. Preliminary analysis suggests that family members participate in many ways in the care of hospitalized older adults including giving information about patients, preventing and managing delirium, providing hands-on care, advocating for patients, aiding in decision-making, and planning for discharge. The quality of existing qualitative research on family involvement during hospitalizations of older adults varies widely. Most existing evidence comes from countries outside of the US. Ultimately, our findings will contribute to the refinement of a conceptual model that will establish a shared language for future multidisciplinary work, inform future intervention development, and support workflow and policy changes.

